# When Standards Meet Reality: An Inverted PORTEC-3 Protocol for High-Risk Endometrial Cancer in Resource-Limited Settings †

**DOI:** 10.3390/cancers18030415

**Published:** 2026-01-28

**Authors:** Raouia Ben Amor, Ines Mlayeh, Amal Riahi, Zeineb Naimi, Myriam Saadi, Rihab Haddad, Ghada Bouguerra, Awatef Hamdoun, Lilia Ghorbel, Nesrine Mejri Turki, Lotfi Kochbati

**Affiliations:** 1 Radiation Oncology Departement, Abderrahmen Mami Hospital, Ariana 2080, Tunisia; 2 Faculty of Medecine of Tunis, Tunis El Manar University, Tunis 1006, Tunisia; 3 Medical Oncology Departement, Abderrahmen Mami Hospital, Ariana 2080, Tunisia

**Keywords:** endometrial cancer, high-risk disease, adjuvant chemoradiotherapy, treatment sequencing, radiotherapy access, disease recurrence, bone marrow sparing, hematologic toxicity

## Abstract

**Background/Objectives:** The combination of chemotherapy and radiotherapy represents a standard adjuvant treatment for patients with high-risk endometrial cancer. However, limited access to radiotherapy in many healthcare systems frequently results in treatment delays, potentially compromising outcomes. The aim of this study was to evaluate the oncologic outcomes and toxicity profile of an inverted treatment sequence consisting of upfront chemotherapy followed by concurrent chemoradiotherapy. **Methods:** We conducted a retrospective single-center study including patients with non-metastatic high-risk endometrial cancer. Eligible patients had FIGO stage I grade 3 disease with lymphovascular space invasion, stage II–III disease, or non-endometrioid histology. All patients received four cycles of paclitaxel–carboplatin followed by pelvic radiotherapy with concurrent cisplatin. Survival outcomes, including local recurrence-free survival, disease-free survival, metastasis-free survival, and overall survival, were analyzed using the Kaplan–Meier method and Cox proportional hazards models. Acute hematologic toxicity was graded according to CTCAE v5.0. Bone marrow dose–volume parameters were evaluated, and receiver operating characteristic curve analysis was performed to identify thresholds associated with grade ≥ 2 hematologic toxicity. **Results:** Fifty-two patients were included, with a median follow-up of 31.4 months. Five-year overall survival and disease-free survival rates were 86.1% and 77.5%, respectively. Ten patients relapsed, with distant metastases observed in all cases and synchronous local recurrence in one. Delays between surgery and radiotherapy of 20 weeks or more, as well as delays exceeding 10 weeks before initiation of chemotherapy, were associated with significantly reduced disease-free survival. Grade ≥ 2 hematologic toxicity was frequent, and neutropenia was associated with inferior overall survival. Bone marrow dose–volume thresholds predictive of hematologic toxicity included V40 Gy < 20–25% and V30 Gy < 40%. **Conclusions:** A chemotherapy-first adjuvant strategy provides favorable oncologic outcomes and excellent locoregional control in high-risk endometrial cancer when radiotherapy is delayed. However, increased hematologic toxicity highlights the importance of optimized bone marrow sparing.

## Simple Summary

Adjuvant management of high-risk endometrial cancer typically combines chemotherapy and radiotherapy; however, timely access to radiotherapy remains inconsistent in many healthcare systems, resulting in significant treatment delays. In this study, we evaluated a chemotherapy-first sequence, followed by combined chemotherapy and radiotherapy, as a pragmatic alternative when radiotherapy cannot start promptly. Survival outcomes, patterns of failure, and treatment-related toxicity were analyzed in patients treated with this adapted approach. As disease recurrence in this population is predominantly metastatic, it was postulated that early initiation of systemic chemotherapy might actually improve metastasis-free survival by targeting occult disease at an earlier stage. Our findings demonstrated excellent locoregional control and favorable survival outcomes when systemic therapy is delivered early, supporting the feasibility of this strategy in routine clinical practice. However, increased hematologic toxicity was observed, emphasizing the importance of optimized treatment planning. We identified bone marrow dose parameters associated with hematologic toxicity, and formulated practical guidance to improve treatment tolerability in real-world settings with limited radiotherapy access.

## 1. Introduction

Endometrial cancer (EC) is the sixth most common malignancy in women, with more than 420,000 new cases reported worldwide in 2022. It predominantly affects postmenopausal women, with a mean age at diagnosis of approximately 60 years [1].

The 2016 ESMO/ESGO/ESTRO consensus classification, updated in 2021, stratifies non-metastatic EC into four risk groups [2,3]. Around 15% of patients are classified as high-risk, defined as stage I endometrioid tumors grade 3 with lymphovascular space invasion (LVSI), stage II or III endometrioid carcinomas, or any stage with non-endometrioid histology such as serous, clear cell, papillary carcinoma, or carcinosarcoma. Histology in this subgroup confers a significantly higher risk of distant metastases and cancer-related mortality [4].

For several decades, pelvic radiotherapy (RT) alone was the standard adjuvant treatment for high-risk EC. However, the PORTEC-3 phase III randomized trial, published in 2018, demonstrated that chemoradiotherapy (CRT) consisting of RT with concurrent cisplatin, followed by adjuvant carboplatin–paclitaxel, improved failure-free survival compared with RT alone and conferred a significant overall survival benefit in stage III disease and non-endometrioid histology [4]. Since then, total abdominal hysterectomy (TAH) with bilateral salpingo-oophorectomy (BSO) and pelvic and para-aortic lymphadenectomy followed by CRT has become the standard of care for this population [3].

In Tunisia, as in many low-resource settings, access to RT is limited, leading to substantial treatment delays. To mitigate these delays, adjuvant chemotherapy is often initiated before CRT while patients await RT scheduling. This “chemo-first” approach is primarily driven by logistical constraints, but it is also supported by a biological rationale; high-risk EC has a high propensity for early distant relapse, and early chemotherapy may reduce micrometastatic burden, potentially lowering the risk of distant failure [5]. Accordingly, we adopted an inverted PORTEC-3 sequence, administering adjuvant chemotherapy first, followed by pelvic RT with concurrent chemotherapy.

The aim of this study was to evaluate local control, local recurrence-free survival (LRFS), disease-free survival (DFS), metastasis-free survival (MFS), overall survival (OS), and treatment-related toxicities in high-risk EC patients treated with this inverted PORTEC-3 protocol.

## 2. Materials and Methods

### 2.1. Study Design and Eligibility Criteria

This retrospective, descriptive, single-center study was conducted between 2018 and 2024 in the Radiation Oncology Department of Abderrahmane Mami Hospital, Ariana, Tunisia. Eligible patients were diagnosed with non-metastatic high-risk endometrial cancer, as defined by the 2016 ESMO/ESGO/ESTRO classification, updated in 2021. High-risk disease was defined as stage I endometrioid carcinoma grade 3 with lymphovascular space invasion (LVSI), stage II–III endometrioid carcinoma of any grade, or tumors of any stage with non-endometrioid histology, including serous, clear cell, papillary carcinoma, or carcinosarcoma.

Only patients with complete pre-treatment staging who were treated according to the inverted PORTEC-3 protocol were included. Additional eligibility criteria required delivery of pelvic external beam radiotherapy (EBRT) to a total dose of 45.0 or 48.6 Gy using either three-dimensional conformal radiotherapy (3D-CRT) or volumetric modulated arc therapy (VMAT), as well as a minimum follow-up of six months after treatment completion.

Patients were excluded if they had incomplete staging, metastatic disease at diagnosis, prior treatment for local recurrence, treatment according to the conventional (non-inverted) PORTEC-3 protocol, treatment discontinuation before completion, or loss to follow-up.

### 2.2. Study Procedures

Clinical, pathological, and technical data were collected retrospectively from medical records and radiotherapy documentation. Recorded variables included demographic characteristics, medical history, imaging findings, pathological features, and FIGO stage.

All patients underwent surgical treatment consisting of total hysterectomy with bilateral salpingo-oophorectomy and pelvic and para-aortic lymphadenectomy. Histopathological data included histologic subtype, tumor grade, depth of myometrial invasion, cervical or extra-uterine extension, number of positive lymph nodes, and LVSI status, categorized as absent, focal (≤5 foci), or extensive (>5 foci).

Adjuvant chemotherapy consisted of four cycles of intravenous paclitaxel (175 mg/m^2^) combined with carboplatin (area under the curve [AUC] 5), administered every 21 days. The number of completed cycles and the interval between surgery and initiation of chemotherapy were recorded for each patient.

Following completion of chemotherapy, all patients underwent CT-based EBRT planning. Patients treated between 2018 and 2022 received 3D-CRT, whereas those treated thereafter received VMAT with image-guided radiotherapy (IGRT).

The prescribed pelvic dose was 45.0 or 48.6 Gy delivered in daily fractions of 1.8 Gy, five days per week. Target volumes, organs at risk (OARs), dosimetric parameters, and treatment intervals were systematically recorded.

Concurrent chemotherapy was cisplatin (50 mg/m^2^) administered during weeks 1 and 4 of EBRT. Carboplatin (AUC 5) was used in patients with contraindications to cisplatin. Following concurrent chemoradiotherapy, all patients received a high-dose-rate (HDR) vaginal brachytherapy boost of 8 Gy delivered in two fractions.

Pelvic bones were contoured as a surrogate for bone marrow, extending from the inferior border of the ischial tuberosities to 25 mm above the planning target volume (PTV). This method is commonly used in radiotherapy studies due to the practical limitations of directly delineating active bone marrow on planning CT scans. It provides an indirect estimate of bone marrow exposure, although it does not capture inter-patient variability in functional bone marrow distribution.

For treatment plan evaluation, the following consensus bone marrow dose constraints were applied, defined as the percentage of the contoured volume receiving at least a specified dose—V10 Gy ≤ 90%, V20 Gy ≤ 75%, and V40 Gy ≤ 37%—where, for example, V20 Gy represents the volume of bone marrow receiving 20 Gy or more.

Hematologic toxicities were graded according to the Common Terminology Criteria for Adverse Events (CTCAE), version 5.0.

### 2.3. Follow-Up and Outcome Definitions

Follow-up was conducted prospectively. All patients underwent a standardized clinical and gynecological evaluation six months after treatment completion. Subsequent follow-up visits were scheduled every three months during the first two years, every six months for the next three years, and annually thereafter. Imaging studies (ultrasound, pelvic MRI, or CT scan) were performed when clinically indicated.

Acute toxicities were prospectively recorded during weekly radiotherapy visits and included hematologic (anemia, neutropenia, lymphopenia, thrombocytopenia), genitourinary (non-infectious cystitis), gastrointestinal (enterocolitis), and vaginal toxicities. Late toxicities included urinary frequency, abdominal bloating, and genital mucosal changes. All adverse events were graded according to CTCAE version 5.0.

Time intervals were defined as follows. The surgery-to-chemotherapy interval was calculated from the date of definitive surgery to the start of adjuvant chemotherapy. The chemotherapy-to-radiotherapy interval was calculated from the end of the last chemotherapy cycle to the initiation of pelvic radiotherapy. The surgery-to-radiotherapy interval was defined as the time from surgery to the start of pelvic radiotherapy. These definitions were applied consistently throughout the analysis.

Overall survival (OS) was defined as the time from diagnosis to death from any cause. Disease-free survival (DFS) was defined as the time from diagnosis to local or distant recurrence, disease progression, or death. Local recurrence-free survival (LRFS) was defined as the time to histologically confirmed vaginal recurrence. Metastasis-free survival (MFS) was defined as the time from diagnosis to distant recurrence.

### 2.4. Statistical Analysis

Statistical analyses were performed using SPSS software (version 27.0). The normality of quantitative variables was assessed using the Kolmogorov–Smirnov test. Quantitative variables were reported as medians with ranges, and qualitative variables as absolute numbers and percentages.

Comparisons between variables were conducted using Student’s *t*-test, analysis of variance (ANOVA), Chi-square test, or Fisher’s exact test, as appropriate. Survival outcomes were estimated using the Kaplan–Meier method, and prognostic factors were evaluated using Cox proportional hazards regression models in univariable and multivariable analyses. A *p*-value < 0.05 was considered statistically significant.

Receiver operating characteristic (ROC) curve analysis was performed to assess the association between bone marrow dose–volume parameters (V10 Gy, V20 Gy, V30 Gy, and V40 Gy) and grade ≥ 2 hematologic toxicities. Logistic regression models were used to evaluate factors associated with binary clinical and dosimetric outcomes. Variables included in multivariable analyses were selected based on clinical relevance and results of univariable analyses, and potential confounders were considered to ensure model robustness. Model performance was assessed using ROC curves, with the area under the curve (AUC) used to evaluate discriminative ability. Optimal cut-off values were determined, and statistical significance was defined as *p* < 0.05.

## 3. Results

### 3.1. Patient Characteristics

**Figure 1:**
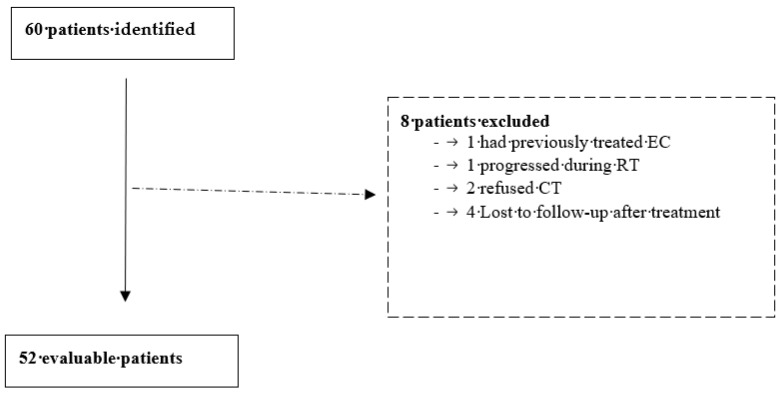
Flow chart.

Between January 2018 and January 2024, 60 patients were enrolled. After exclusion, a total of 52 patients were evaluable (Figure 1). All patients were female (100%).

Patient characteristics are summarized in Table 1.

Median age was 63 years (IQR 37–76), with 21% (n = 11) over 70 years. Most patients were overweight (BMI ≥ 25, 75%, n = 39), and 38% (n = 20) were obese (BMI ≥ 30).

Histopathological analysis revealed that 63% (n = 33) had endometrioid endometrial cancer (type 1), while aggressive histologies, including grade 3 type 1 and type 2 EC, accounted for 37% (n = 19). LVSI was present in 65% (n = 34), equally distributed between focal and extensive. FIGO 2018 stage III disease was observed in 71.2% (n = 37), including stage IIIC1 in 30.8% (n = 16) and IIIC2 in 23.1% (n = 12).

All patients received adjuvant paclitaxel–carboplatin chemotherapy, with most completing 4 cycles (92.3%, n = 48). The median interval from surgery to chemotherapy was 11 weeks (range, 3.6–31 weeks). Adjuvant radiotherapy was started after a median of 26 weeks post-surgery and 8.6 weeks post-chemotherapy. Techniques included 3D conformal RT (62%, n = 32) and VMAT (38%, n = 20), with prescription doses of 45 Gy in 25 fractions (90%, n = 47) or 48.6 Gy in 27 fractions (10%, n = 5); 19.2% (n = 10) received latero-aortic lymph node irradiation.

A brachytherapy boost was delivered to 71% (n = 37) of patients (8 Gy in 2 fractions). Concurrent chemotherapy (CT) was administered with radiotherapy (RT) in 77% of patients (n = 40). Cisplatin was used in most cases (95%, n = 38), while carboplatin was given to 5% of patients (n = 2) with impaired renal function. Regarding the number of concomitant CT cycles, 8 patients received 1 cycle, 29 patients received 2 cycles, 1 patient received 3 cycles, and 2 patients received 5 cycles.

### 3.2. Outcomes

After a median follow-up of 31.4 months (range, 8.3–81.8), 10 recurrences and 6 deaths were observed, with 90% of events occurring within the first two years post-treatment. Recurrences occurred at distant sites in all 10 cases, with synchronous local recurrence in 1 case. Detailed oncologic outcomes are summarized in Table 2.

**Figure 2:**
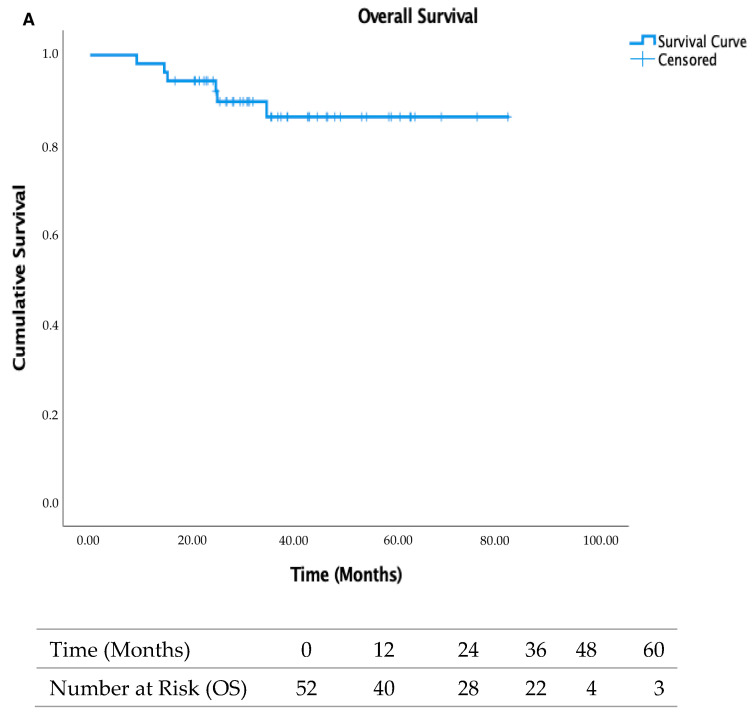

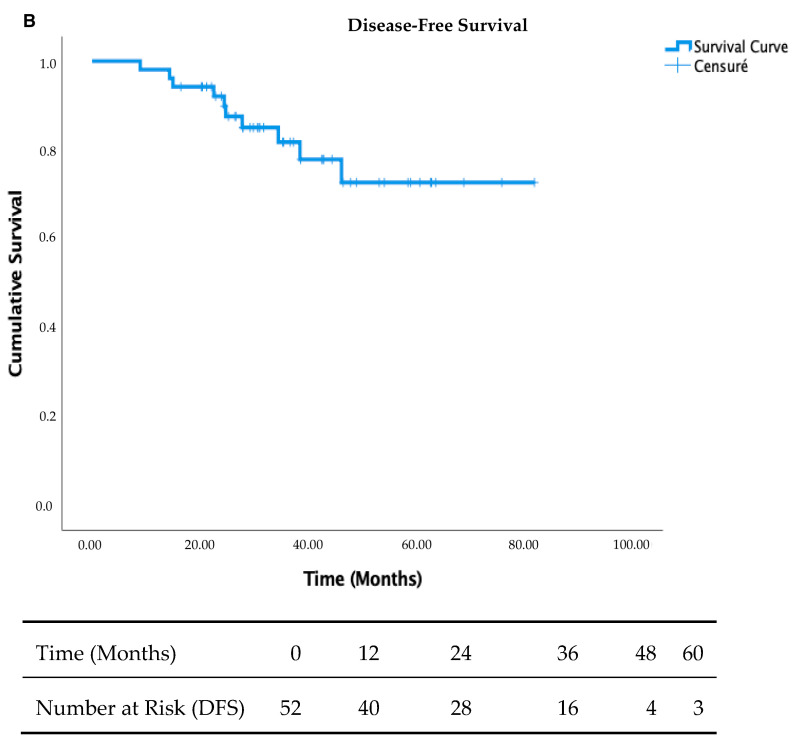
Overall survival (OS, panel (**A**)) and disease-free survival (DFS, panel (**B**)) in the study cohort.

The 5-year overall survival (OS) was 86.1%, while 5-year disease-free survival (DFS), local recurrence-free survival (LRFS), and metastasis-free survival (MFS) were 77.5%, 97.9%, and 79.3%, respectively (Figure 2).

### 3.3. Univariable and Multivariable Analyses of Prognostic Factors

Obesity (BMI ≥ 30) was associated with significantly lower disease-free survival (DFS), with 5-year DFS rates of 64.4% compared with 88.3% in patients with BMI < 30 (HR = 5.43, 95% CI 1.15–5.64, *p* = 0.033). Smoking status was also associated with DFS, with 5-year DFS rates of 29.6% in smokers versus 90.6% in non-smokers (HR = 4.88, 95% CI 2.21–8.06, *p* = 0.001).

Aggressive histology (grade 3 endometrioid or non-endometrioid carcinoma) was associated with lower overall survival (OS) (5-year OS: 77.4% vs. 100%; HR = 4.55, 95% CI 1.57–9.83, *p* = 0.042) and DFS (70% vs. 87.5%; HR = 3.49, 95% CI 1.82–7.29, *p* = 0.041).

Lymphovascular space invasion (LVSI) was not significantly associated with OS (*p* = 0.074) but was associated with DFS. Five-year DFS rates were 100% in patients without LVSI, 74.1% in those with focal LVSI, and 63% in those with extensive LVSI (HR = 3.6, 95% CI 1.17–10.04, *p* = 0.025).

FIGO stage III disease was associated with lower survival outcomes. Five-year OS was 70.8% for stage III compared with 100% for stages I–II (HR = 3.62, 95% CI 1.8–8.39, *p* = 0.031). Similarly, 5-year DFS was 53.8% for stage III compared with 95.8% for stage II and 100% for stage I (HR = 3.31, 95% CI 1.45–6.17, *p* = 0.021). OS and DFS according to clinicopathological variables are summarized in Table 3.

### 3.4. Treatment-Related Outcomes

The number of adjuvant paclitaxel–carboplatin cycles (three vs. four or six) was not significantly associated with OS or DFS (*p* = 0.2). Radiotherapy dose (45 Gy vs. 48.6 Gy) and the use of a brachytherapy boost were also not associated with differences in OS or DFS (*p* = 0.9 and *p* = 0.08).

The radiotherapy technique was associated with differences in DFS. Patients treated with VMAT had higher DFS compared with those receiving 3D conformal radiotherapy (5-year DFS: 100% vs. 73.9%; HR = 0.48, 95% CI 0.10–0.30, *p* = 0.036).

Concurrent chemotherapy, analyzed as a binary variable, was not significantly associated with OS or DFS (*p* = 0.06). However, receiving more than two weekly concurrent cycles was associated with lower survival outcomes. Five-year OS was 33.3% in patients receiving more than two cycles compared with 91.2% in those receiving one or two cycles (HR = 15.04, 95% CI 2.11–107.33, *p* = 0.007). Similarly, 5-year DFS was 33.3% versus 80.1%, respectively (HR = 7.89, 95% CI 1.47–42.24, *p* = 0.016).

Treatment time intervals were associated with DFS. DFS rates were higher in patients with a surgery-to-chemotherapy interval ≤ 10 weeks, a chemotherapy-to-radiotherapy interval ≤ 6 weeks, and a surgery-to-radiotherapy interval ≤ 20 weeks (Table 4). These time interval categories were not significantly associated with OS.

In multivariable analysis, obesity (HR = 4.80; 95% CI 1.64–5.20; *p* = 0.018), surgery-to-radiotherapy interval > 20 weeks (HR = 4.49; 95% CI 1.77–6.11; *p* = 0.020), and surgery-to-chemotherapy interval > 10 weeks (HR = 2.10; 95% CI 1.06–4.19; *p* = 0.034) were independently associated with decreased DFS. None of these factors significantly affected OS (Table 5).

### 3.5. Treatment-Related Toxicities

Acute treatment-related toxicities were predominantly hematologic. Anemia was observed in 44.2% of patients, lymphopenia in 75%, neutropenia in 32.7%, and thrombocytopenia in 25%. Grade ≥ 2 hematologic toxicity occurred in 67.3% of cases. Acute gastrointestinal toxicity affected 53.8% of patients, with grade ≥ 3 events in 3.8%. Non-infectious cystitis and vaginal inflammation were reported in 42.3% and 34.6% of patients, respectively, with no grade ≥ 3 genitourinary toxicities observed (Table 6).

All documented late toxicities were grade 1.

Acute hematologic toxicities were reported with corresponding DFS and OS outcomes. Lymphopenia was associated with lower DFS (5-year DFS: 68% vs. 100%; HR = 3.83, 95% CI 1.11–11.37, *p* = 0.04). Neutropenia was associated with lower OS (60.4% vs. 96.4%; HR = 2.96, 95% CI 1.50–6.96, *p* = 0.02) and lower DFS (64.7% vs. 84.5%; HR = 2.23, 95% CI 1.18–5.21, *p* = 0.027). Thrombocytopenia showed trends toward lower OS (*p* = 0.142) and DFS (*p* = 0.051). Other acute toxicities, including enterocolitis, non-infectious cystitis, erythroderma, and vaginal inflammation, were not associated with statistically significant differences in OS or DFS. Detailed survival analyses according to toxicity profiles are provided in Supplementary Table S1.

### 3.6. Bone Marrow Dosimetric Parameters

An analysis of bone marrow dosimetric parameters revealed a statistically significant association between the maximum bone marrow dose (Dmax) and the occurrence of lymphopenia (*p* = 0.035). The remaining dosimetric parameters, including Dmean and the volume percentages receiving 5 Gy, 10 Gy, 20 Gy, 30 Gy, and 40 Gy, did not demonstrate significant correlations with the different hematologic toxicities (Table 7).

**Figure 3:**
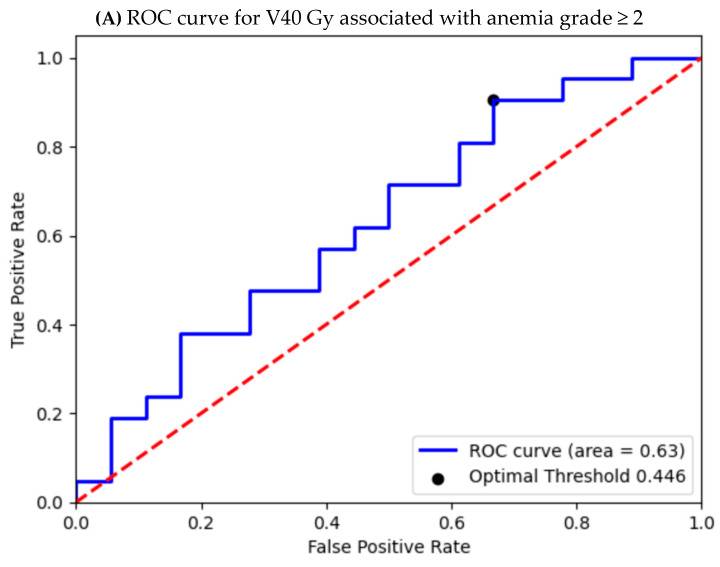

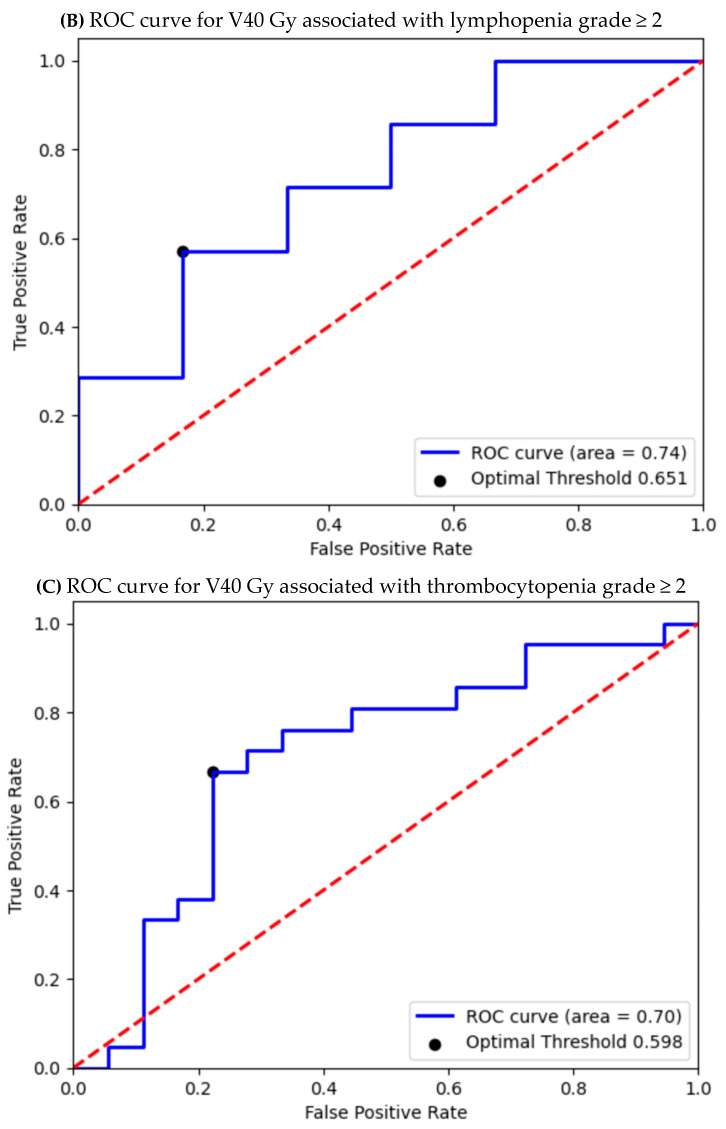

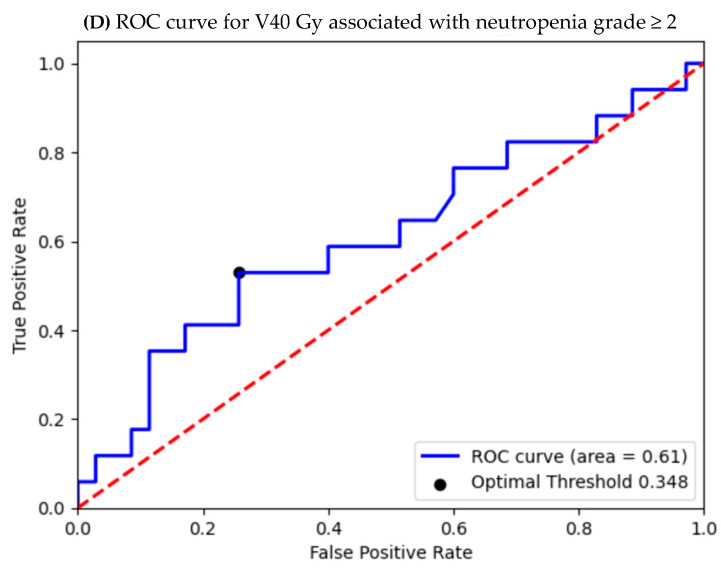
ROC curve of sensitivity versus specificity of V 40 Gy in (**A**) anemia grade ≥ 2, (**B**) lymphopenia grade ≥ 2, (**C**) thrombocytopenia grade ≥ 2, and (**D**) neutropenia grade ≥ 2 toxicities.

Selected bone marrow dose–volume cut-off values demonstrated discriminatory ability for predicting grade ≥2 hematologic toxicities. For lymphopenia, significant cut-off values were identified for V20Gy ≥ 78.7% (*p* = 0.057), V30Gy ≥ 36.16% (*p* = 0.011), and V40Gy ≥ 22.78% (*p* = 0.009). For grade ≥ 2 anemia, significant cut-off values were observed for V20Gy ≥ 83.1% (*p* = 0.002), V30Gy ≥ 40.6% (*p* = 0.030), and V40Gy ≥ 31.27% (*p* = 0.019). Grade ≥ 2 neutropenia was associated with V30Gy ≥ 44.4% (*p* = 0.036) and V40Gy ≥ 34.64% (*p* = 0.025). For grade ≥ 2 thrombocytopenia, only V40Gy ≥ 20.35% reached statistical significance (*p* = 0.030) (Figure 3; Table 8).

## 4. Discussion

In this cohort of high-risk endometrial cancer (EC), we observed a 5-year overall survival (OS) of 86.1%, disease-free survival (DFS) of 77.5%, local recurrence-free survival (LRFS) of 97.9%, and metastasis-free survival (MFS) of 79.3%. These figures compare well with landmark trials; for instance, PORTEC-3 reported a 5-year OS of ~81.8% in its chemoradiotherapy (CRT) arm [4], and the RTOG-9708 phase II study noted a 4-year OS of ~85% [6].

### 4.1. Patient Characteristics and Risk Context

The patient characteristics in our study closely mirror those in PORTEC-3. In our study, the median age was 63 years; 63.5% had endometrioid histology and 65.4% presented with lymphovascular space invasion (LVSI). This is compared to a median age of 62, where 66% had endometrioid histology and the presence of LVSI was 60% in PORTEC-3 [4]. Focal and extensive LVSI were each observed in 32.7% of our patients, which aligns with the ESGO/ESTRO/ESP 2021 risk stratification guidelines [3]. Stage III disease was more prevalent in our cohort (53.9%) than in PORTEC-3 (46%), underscoring the high-risk nature of the population [4].

Local recurrence in EC typically involves the vaginal cuff or central pelvis [7,8]. The literature reports vaginal relapse rates between 2–11%, usually occurring around 22 months post-treatment, with central pelvic recurrences being less common (~3%) [7,8]. When isolated, vaginal recurrences carry a favorable prognosis; 5-year OS in such cases has been reported at ~77% [9]. In PORTEC-3, isolated vaginal recurrence occurred in <1% of patients per arm [4]. In our cohort, no isolated vaginal relapse was observed; one patient experienced synchronous local and distant failure. The high 5-year LRFS (97.9%) observed suggests that surgical management followed by radiotherapy confers excellent local control, even when radiotherapy is delayed until after chemotherapy [3,4].

Distant metastases remain the most significant threat to survival in high-risk EC. Common metastatic sites include the lung and peritoneum [10]. In our series, 100% of recurrences were metastatic (17.3% of the cohort), with 60% pulmonary and 40% peritoneal involvement; rates slightly lower than those reported in PORTEC-3 (22–28%) [4,10]. These data reinforce the concept that systemic disease is the primary driver of long-term outcome, highlighting the need for aggressive systemic therapy [11,12,13].

Given the prevalence of distant relapse, optimizing the sequencing of therapy is imperative. Our data suggest that administering chemotherapy before radiotherapy may improve systemic control and DFS. Notably, patients in our cohort who completed their treatment within optimal time intervals had a 5-year DFS of 95.2%, marking a substantial improvement over the overall average. This finding supports the potential efficacy of a chemotherapy-first strategy in high-risk EC, a concept corroborated by other studies in FIGO IIIC disease [14,15,16].

While the median follow-up in our study is relatively limited at 31 months, a substantial portion of EC recurrences occur within the first two years of follow-up, a period covered by our current data. Nonetheless, a longer follow-up is necessary to confirm the sustainability of this survival advantage.

The chemotherapy-first approach offers several practical advantages, which are supported by clinical evidence. In a cohort of women with FIGO IIIC EC, those who received six cycles of chemotherapy prior to radiotherapy had a 3-year OS of 81.7%, compared to 70.3% in a “sandwich” regimen (three cycles → RT → three cycles) [15]. Importantly, 94.9% of patients in the chemo-first group completed four or more cycles. The same study’s multivariate analysis found that ≥4 chemotherapy cycles were associated with a lower rate of distant relapse compared with three cycles, indicating a dose–response relationship [15,16,17].

The benefit of CRT versus RT alone is well-established in randomized trials. In PORTEC-3, CRT significantly reduced distant relapse (5-year risk 21.4% vs. 29.1%; HR = 0.74; *p* = 0.047) and improved failure-free survival [4]. Long-term follow-up further supported these benefits; a 10-year analysis reported OS of 74.4% for CRT versus 67.3% for RT alone (HR = 0.73; *p* = 0.032), and recurrence-free survival of 72.8% vs. 67.4% (HR = 0.74; *p* = 0.034) [4]. Meta-analyses and real-world data further support CRT as standard-of-care in high-risk EC, with tolerable toxicity [14,18,19,20].

In our cohort, a small subset of patients (5%) received more than two concurrent chemotherapy cycles following a weekly cisplatin schedule, prior to the adoption of PORTEC-3 guidelines. These patients experienced worse oncologic outcomes, which may reflect increased toxicity and treatment disruption. This observation is consistent with previous reports, as weekly concurrent chemotherapy has been associated with higher rates of acute toxicity; for example, Jakubowicz et al. (2014) reported grade 3–4 toxicity in 21.6% of patients and treatment interruptions in 20% when weekly cisplatin was combined with pelvic radiotherapy [21].

Our findings advocate for a chemotherapy-first adjuvant approach, particularly valuable in real-world settings where radiotherapy access may be delayed. Administering systemic therapy early targets micrometastatic disease, permits full-dose chemotherapy delivery before potential bone marrow suppression from RT, and maximizes chemotherapy completion rates [17,22,23]. This approach may also mitigate the negative effects of prolonged intervals between surgery and RT, which adversely affect outcomes [24,25].

Retrospective data comparing sandwich versus sequential chemoradiotherapy in stage III EC (or IIIC) show favorable outcomes for sandwich sequencing in terms of disease-specific survival and local control [16,26,27]. In one meta-analysis including 800 patients, sandwich therapy was associated with improved 5-year OS compared with sequential therapy, particularly in non-endometrioid histologies, without a major increase in toxicity [16,28,29].

McEachron et al. found that the optimal sequence (chemo → RT → chemo) was associated with significantly better 3-year PFS and OS (*p* < 0.001) compared to RT-first regimens in advanced EC (n = 152) [5]. This supports the feasibility of different sequencing strategies tailored to patient and institutional constraints.

Current advances in EC management emphasize molecular classification, including *POLE* (ultramutated), Mismatch Repair (MMR) status, and p53 status, which are critical for risk stratification. While comprehensive POLE status was not universally available in our cohort (due to the necessity of molecular sequencing), immunohistochemistry (IHC) for p53 and MSI was generally accessible. The latest updates from the PORTEC-3 trial have established that the primary benefit of CRT, particularly regarding OS, is concentrated within the p53 abnormal (p53abn) subgroup, which is associated with the highest systemic recurrence risk. Given the high systemic failure rate in this subgroup, our findings advocating for a chemotherapy-first sequence are particularly pertinent. This inverted sequencing may offer maximal systemic benefit upfront for the p53 abnormal group, where systemic control is paramount [30,31].

### 4.2. Safety Profile and Novel Bone Marrow Dose Constraints

CRT remains more toxic than RT alone, but the toxicity profile is manageable. Meta-analyses report higher acute grade III/IV toxicities with CRT, with no clear increase in late toxicity [14,19,32].

While CRT is associated with manageable toxicity, it remains myelosuppressive. The high incidence of Grade 3–4 cytopenias observed in PORTEC-3 (45%) was mirrored in our cohort, particularly for lymphopenia (40.4%), neutropenia (11.5%), thrombocytopenia (9.6%), and anemia (5.8%). Crucially, this myelotoxicity was clinically relevant, with neutropenia, lymphopenia, and thrombocytopenia all significantly correlating with reduced survival endpoints [4]. This corroborates evidence suggesting that high-grade myelosuppression may compromise treatment efficacy.

To mitigate this risk, we analyzed dose–volume parameters and identified specific, stringent thresholds predictive of Grade 2 cytopenias across all lineages. ROC curve analyses demonstrated AUC values ranging from 0.6 to 0.88, indicating acceptable to excellent discriminative ability for these thresholds. The identified thresholds were notably stricter than the V40 Gy ≤ 37% constraint recommended by RTOG-1203 [33]. This disparity is likely due to the increased susceptibility of our patient population, who had received adjuvant chemotherapy prior to pelvic radiotherapy, highlighting the synergistic myelosuppressive effect of this treatment sequencing.

Based on these original dosimetric findings, we propose a mandatory tightening of pelvic bone marrow constraints: specifically, lowering the recommended V40 Gy to 20–25% and introducing a new constraint to maintain V30 Gy below 40–45%. These revised constraints are essential to mitigate against severe hematologic toxicity and preserve oncologic outcomes in patients with prior chemotherapy exposure.

### 4.3. Strengths, Limitations, and Future Directions

The primary strength of our study lies in the demonstration that a chemotherapy-first strategy is feasible, well tolerated and effective in a real-world high-risk EC cohort. Limitations include its retrospective design, relatively small sample size, which limits statistical power and precludes a full multivariable analysis, unicentric design, potential selection bias, and the lack of systematic comorbidity assessment. Comprehensive molecular classification (e.g., p53, MMR, POLE status) was unavailable for all patients, which may impact risk stratification and therapeutic tailoring [3,30].

Prospective trials are required to compare chemotherapy-first versus conventional sequencing definitively, ideally stratified by molecular subtype. Efforts should also be made to minimize delays to radiotherapy, particularly in resource-constrained settings, to optimize outcomes [3,4,15,16].

## 5. Conclusions

In high-risk endometrial cancer, where distant metastasis is the main determinant of survival, an adjuvant chemotherapy-first strategy provides a rational therapeutic priority. This approach enables early systemic therapy, maximizing cytotoxic delivery to target micrometastatic disease. In our study, we demonstrated that this strategy was associated with favorable oncologic outcomes and excellent locoregional control despite radiotherapy being delayed. Importantly, however, our analysis demonstrated that prior chemotherapy increased pelvic bone marrow radiosensitivity, requiring stricter dosimetric limits than current guidelines recommend. To reduce treatment-related hematologic toxicity in future cases, we propose specific constraints: V40 Gy ≤ 20–25% and V30 Gy < 40–45%.

These findings support the prospective evaluation of tailored sequencing strategies and the incorporation of patient-specific, bone marrow-sparing radiotherapy planning to optimize the therapeutic index in this high-risk population.

## Figures and Tables

**Table 1:** Patient, disease, and treatment characteristics.

Characteristics	Number of Patients (N)	Percentage (%)
Medical history		
Breast cancer	1	1.9
Hypertension	26	50
Diabetes	19	36.5
Obesity	20	38.5
Smoking history	9	17.3
FIGO 2018 stage		
IA	3	5.8
IB	7	13.4
II	5	9.6
IIIA	8	15.4
IIIB	1	1.9
IIIC1	16	30.8
IIIC2	12	23.1
Histological type		
Endometrioid	33	63.5
Serous	6	11.5
Carcinosarcoma	7	13.5
Mixed	2	3.8
Mucinous	1	1.9
Clear cell	3	5.8
Histological grade		
Grade 1 or 2	27	51.9
Grade 3	25	48.1
Aggressive histological type (Type 1 grade 3 or Type 2)	32	61.5
LVSI		
Absent	18	34.6
Present and focal	17	32.7
Present and extensive	17	32.7
Surgery		
TAH/BSO	28	53.8
TAH/BSO + omentectomy + peritoneal cytology	24	46.2
Lymph node assessment		
Full staging	31	59.6
Pelvic lymphadenectomy	17	32.7
Sentinel lymph node	1	1.9
No lymph node assessment	3	5.8
Adjuvant chemotherapy courses	52	100
3 cycles	1	1.9
4 cycles	48	92.3
6 cycles	3	5.8
Radiotherapy		
Three-dimensional technique	32	61.5
VMAT technique	20	38.5
Prescription dose = 45 Gy	47	90.4
Prescription dose = 48.6 Gy	5	9.6
Brachytherapy boost	37	71.2
Concurrent chemotherapy	40	76.9
Cisplatin	38	95
Carboplatin	2	5
Number of cycles		
1 cycle	8	5
2 cycles	29	72.5
3 cycles	1	2.5
5 cycles	2	5

**Table 2:** Sites of recurrence.

Site of Recurrence	Number of Patients	Percentage (%)
Local (vaginal cuff)	0	0
Pelvic lymph nodes	1	10
Peritoneal carcinomatosis	4	40
Distant (lung)	4	40
Distant (lung) + Local (vaginal cuff)	1	10

**Table 3:** Univariable analysis for histoprognostic factors for OS and DFS.

Histology Findings		5-Year OS	HR	95% CI	*p*-Value	5-Year DFS	HR	95% CI	*p*-Value
Histological type	Type 1	85.3%	0.92	0.16–5.03	0.924	73.1%	0.78	0.20–3.02	0.719
	Type 2	88%				83.9%			
Grade	1 or 2	96.3%	5.68	0.66–8.65	0.113	87.5%	4.784	1.01–8.55	0.048
	3	75.2%				66%			
Aggressive histology	EEC * grade 1 or 2	100%	4.55	1.57–9.83	0.042	87.5%	3.49	1.82–7.29	0.041
	EEC grade 3 or non-EEC *	77.4%				70%			
FIGO Stage	Stage I	100%	3.62	1.8 –8.39	0.031	100%	3.31	1.45–6.17	0.021
	Stage II	100%				95.8%			
	Stage III	70.8%				53.8%			
LVSI *	Focal	94.4%	5.69	0.84–8.27	0.074	74.1%	3.6	1.17–7.04	0.025
	Extensive	70.9%				63%			
	Absent	100%				100%			

* EEC = endometroid endometrial carcinoma; LVSI = lymphovascular space invasion; HR = hazard ratio; CI = confidence interval.

**Table 4:** Univariable analysis of treatment-related prognostic factors for OS and DFS.

Variables		OS 5-Years	HR	95% IC	*p*-Value	DFS 5-Years	HR	95% IC	*p*-Value
Delays between surgery-CT *	≤10 weeks	100%	3.41	0.39–29.25	0.263	95.2%	1.77	1.85–5.57	**0.036**
	˃10 weeks	80%				64.7%			
Delays between CT-RT *	≤6 weeks	93.8%	3.30	0.38–11.28	0.275	95%	2.45	1.82–6.04	**0.042**
	˃6 weeks	81.4%				65.3%			
Delays between surgery-RT *	<20 weeks	95.2%	6.50	0.8–12.06	0.279	95.2%	3.27	1.38–5.40	**0.041**
	≥20 weeks	80.7%				65.7%			
Delays between RT-BT *	<2 weeks	100%	4.60	0.9–11.01	0.393	100%	3.87	1.04–11.49	0.3
	≥2 weeks	78.8%				69.7%			

* CT = chemotherapy; RT = radiotherapy; BT = brachytherapy; OS = overall survival; DFS = disease-free survival; HR = hazard ratio; CI = confidence interval.

**Table 5:** Multivariable analysis of factors independently predicting reduced disease-free survival (DFS).

Factor	HR *	95% CI *	*p*-Value
Obesity (yes)	4.80	1.64	0.018
Aggressive histologic subtype (yes)	2.55	0.64–10.17	0.19
Stage II	0.56	0.12–2.60	0.46
Stage III	1.86	0.41–8.55	0.42
Stages I + II (vs. III)	0.55	0.12–2.57	0.45
Para-aortic irradiation (yes)	2.80	0.64–12.20	0.17
Concurrent CT cycles > 2	2.49	0.66–9.31	0.18
Neutropenia (yes)	2.80	0.77–10.11	0.12
Surgery-to-radiotherapy interval > 20 weeks	4.49	1.77–6.11	0.02
Surgery-to-chemotherapy interval > 10 weeks	2.10	1.06–4.19	0.034

* HR = hazard ratio; CI = confidence interval.

**Table 6:** Acute treatment-related toxicities graded according to CTCAE version 5.0.

Acute Toxicities	Number of Patients (N)	Percentage (%)	Grade 1–2	Grade 3–4	*p*
Anemia	23	44.2	20	3	0.00
Lymphopenia	39	75	18	21	0.04
Neutropenia	17	32.7	11	6	0.02
Thrombocytopenia	13	25	8	5	0.10
Enterocolitis	28	53.8	26	2	0.03
Urinary	22	42.3	22	0	0.00
Vaginal inflammation	18	34.6	18	0	0.00

**Table 7:** Association between bone marrow dosimetric parameters and hematologic toxicities.

Dosimetric Parameter	Anemia	Neutropenia	Lymphopenia	Thrombocytopenia
Dmean *	0.648	0.167	0.095	0.579
Dmax *	0.478	0.696	0.035	0.486
V5Gy	0.665	0.226	0.447	0.907
V10Gy	0.549	0.201	0.440	0.949
V20Gy	0.828	0.143	0.129	0.514
V30Gy	0.511	0.413	0.058	0.846
V40Gy	0.691	0.595	0.123	0.933

* Dmean = mean dose; Dmax = maximal dose.

**Table 8:** Comparison of detection of V10 Gy, V30 Gy, V20 Gy, and V40 Gy on grade ≥ hematological toxicities.

	Cutt-Off Value (%)	Accuracy	Sensitivity	Specificity	Roc_Auc *	*p*
Anemia Grade ≥ 2						
V10 Gy	83.684	0.696	0.444	0.857	0.437	0.012
V20 Gy	83.100	0.696	0.444	0.857	0.540	0.002
V30 Gy	40.620	0.609	0.667	0.571	0.571	0.030
V40 Gy	31.270	0.652	0.444	0.786	0.630	0.019
Lymphopenia Grade ≥ 2						
V10 Gy	87.680	0.718	0.667	0.778	0.471	0.132
V20 Gy	78.700	0.667	0.571	0.778	0.546	0.057
V30 Gy	36.166	0.590	0.810	0.333	0.881	0.011
V40 Gy	22.784	0.590	0.667	0.500	0.743	0.009
Neutropenia Grade ≥ 2						
V10 Gy	82.558	0.647	0.400	1.000	0.457	0.062
V20 Gy	70.321	0.706	0.700	0.714	0.550	0.047
V30 Gy	44.400	0.647	0.500	0.857	0.823	0.036
V40 Gy	34.640	0.588	0.300	1.000	0.619	0.025
Thrombocytopenia Grade ≥ 2						
V10 Gy	83.684	0.846	1.000	0.667	0.482	0.289
V20 Gy	88.660	0.692	0.429	1.000	0.438	0.114
V30 Gy	40.912	0.769	0.857	0.667	0.714	0.088
V40 Gy	20.350	0.692	0.857	0.500	0.701	0.030

* Roc_Auc = area under the ROC curve.

## Data Availability

The data presented in this study are available on reasonable request from the corresponding author. The data are not publicly available due to privacy and ethical restrictions.

## Supplementary Materials

The following supporting information can be downloaded at https://www.mdpi.com/article/10.3390/cancers18030415/s1: Table S1: Analysis of overall survival and disease-free survival according to acute and late treatment-related toxicities.

## Abbreviations

The following abbreviations are used in this manuscript:

BM	Bone Marrow
CRT	Chemoradiotherapy
CT	Chemotherapy
CTCAE	Common Terminology Criteria for Adverse Events
DFS	Disease-Free Survival
EC	Endometrial Cancer
FIGO	International Federation of Gynecology and Obstetrics
HT	Hematological Toxicity
LRS	Low-Resource Setting
LRFS	Local Recurrence-Free Survival
LVSI	Lymphovascular Space Invasion
MFS	Metastasis-Free Survival
OS	Overall Survival
ROC	Receiver Operating Characteristic
RT	Radiotherapy
V30Gy	Volume Receiving 30 Gray
V40Gy	Volume Receiving 40 Gray
