# Supplementary material for: When Standards Meet Reality: An Inverted PORTEC-3 Protocol for High-Risk Endometrial Cancer in Resource-Limited Settings
Source: Cancers (Basel). 2026 Jan 28;18(3):415. doi: 10.3390/cancers18030415 (PMC12896600; doi:10.3390/cancers18030415)
Supplement: Supplementary file 1 [file cancers-18-00415-s001.zip › cancers-4072329-supplementary.pdf]

Table S1. Analysis of overall survival and disease-free survival based on acute and late toxicities.

Toxicity	5-year OS (%)	HR	95% CI	p-value	5-year DFS (%)	HR	95% CI	p-value
**Anemia**	87	1.41	0.28–6.99	0.675	70.7	1.28	0.37–4.42	0.700
**Lymphopenia**	80.6	5.34	0.02–9,57	0.347	68	3.83	1.11–5.37	**0.040**
**Neutropenia**	60.4	2.96	1.50–6,96	**0.020**	64.7	2.23	1.18–5.21	**0.027**
**Thrombocytopenia**	66.5	3.32	0.67–6.49	0.142	46.2	3.45	1.99–6.98	0.051
**Enterocolitis**	88.2	1.29	0.26–6.37	0.759	67.9	1.87	0.53–6.64	0.333
**Non-infectious cystitis**	80.8	0.35	0.06–1.92	0.226	73.6	1.05	0.30–3.71	0.944
**Erythroderma**	78	0.52	0.11–2.58	0.424	74.4	1.24	0.32–4.78	0.759
**Vaginal inflammation**	88.5	1.21	0.22–6.60	0.829	69.9	2.35	0.50–11.10	0.280

OS *= Overall survival;* ***DFS*** *= Disease-free survival;* ***HR*** *= Hazard ratio;* ***CI*** *= Confidence interval.*
